# Early Intergenerational Relationships and Later Support Provided to Older Parents: Time-to-Death as a Contingency

**DOI:** 10.1093/geroni/igab046.1242

**Published:** 2021-12-17

**Authors:** Merril Silverstein, Wencheng Zhang, Douglas Wolf, Maria Brown

**Affiliations:** 1 Syracuse University, Syracuse, New York, New York, United States; 2 Syracuse University, Syracuse University, New York, United States; 3 Syracuse University, Syracuse, New York, United States

## Abstract

This paper focuses on whether stronger relationships with parents early in the family lifecycle results in adult children providing more support to them 45 years later, and whether this association is contingent on parents’ remaining years of life. We test time-to-death of parents as an indicator of vulnerability, an easy to ascertain and potentially powerful predictor of support. Data derived from the Longitudinal Study of Generations, a panel of three-generation families, originally fielded in 1971 and continuing to 2016. Focusing on the youngest generation (mean age = 19 in 1971), the analytic sample consists of 356 child-father relationships 473 child-mother relationships. We examined trajectories of instrumental support provided to parents over four waves between 1997 and 2016 as a function of each parent’s remaining years of life (mortality data from the National Death Index). We also examined variation in those trajectories based on frequency of shared activities and intensity of emotional closeness in 1971. Ordinal multi-level growth curve analysis revealed that proximity to death was a significant predictor of instrumental support provided over time. Only in child-father relationships did greater emotional closeness, as expressed in 1971, produce stronger associations between remaining years of life and provision of instrumental support. Findings are discussed in terms of understanding intergenerational dynamics that unfold over many decades and the utility of time-to-death as an alternative metric for assessing vulnerability. This research is timely in light of growing uncertainty about the family as a reliable source of care in later life, particularly for older men.

